# Elucidating the Neuropsychiatric Phenomena of Antiphospholipid Syndrome in a 31-Year-Old Female

**DOI:** 10.7759/cureus.64856

**Published:** 2024-07-18

**Authors:** Fares Jamal, Ravina Kumar, Narek Hakobyan

**Affiliations:** 1 Internal Medicine, Brookdale University Hospital Medical Center, Brooklyn, USA

**Keywords:** hypercoagulable, stroke, psychiatric, psychosis, antibodies, anticardiolipin, antiphospholipid

## Abstract

We present an unusual case of antiphospholipid syndrome (APS) in a 31-year-old female patient exhibiting neuropsychiatric manifestations, followed by a subsequent thromboembolic stroke. APS is characterized by antiphospholipid antibodies leading to a prothrombotic state and an increased risk of thrombotic events. While the neurological involvement in APS typically presents with thrombotic events, antiphospholipid antibodies may also directly interact with neural tissue, causing immediate pathogenic effects that disrupt normal function. Neuropsychiatric manifestations in APS are rare but have been documented previously, including cases of psychosis and hallucinations. The timely recognition of APS in patients with neuropsychiatric symptoms is crucial for appropriate management and the prevention of further complications. The reported patient displayed aggressive, bizarre, and erratic behavior upon admission to the psychiatric unit, followed by the development of right-sided facial droop and weakness. Imaging studies revealed stenosis and partial occlusion of the left middle cerebral artery (MCA), and a repeat scan showed a known left MCA territory infarct with increasing hypodensity in specific brain regions. Notably, the patient exhibited multiple purpuric ecchymoses on bilateral upper extremities, raising suspicion of a hypercoagulable state. Laboratory investigations detected elevated levels of anticardiolipin IgG and beta-2 glycoprotein 1 IgG, along with a positive antinuclear antibody. The presence of a patent foramen ovale was also confirmed through echocardiography. This case emphasizes the importance of early APS recognition in patients with neuropsychiatric symptoms, facilitating appropriate intervention and improved outcomes. Further research is warranted to elucidate the underlying pathophysiological mechanisms connecting APS to neuropsychiatric manifestations, enabling enhanced understanding and refined management of this intricate condition.

## Introduction

Antiphospholipid syndrome (APS) is a multisystemic disorder characterized by the development of venous and/or arterial thrombi, recurrent fetal loss, and potential coexistence with mild thrombocytopenia [1]. The pathophysiological mechanisms underlying APS involve the activation of platelets, endothelial cells, monocytes, and the complement system, collectively contributing to a prothrombotic state [2]. The annual incidence of APS is approximately 5 per 100,000 individuals, with a prevalence ranging from 40 to 50 per 100,000 people [2]. APS may present in two primary forms, namely, as a standalone condition not associated with any other autoimmune disease (primary APS) or in conjunction with other autoimmune diseases, notably systemic lupus erythematosus (secondary APS) [3]. The classification of APS as catastrophic occurs when it leads to widespread vaso-occlusion in numerous small vessels within a short period [1]. Furthermore, APS bears significant clinical implications. It accounts for 20%-30% of strokes in individuals younger than 50 years old, contributes to 20% of unprovoked deep vein thromboses, and affects 10%-15% of women experiencing recurrent abortions [3]. Such observations emphasize the importance of understanding and managing APS to prevent severe complications and adverse outcomes in affected individuals.

APS is characterized by antibodies that do not directly interact with phospholipids but instead bind to plasma proteins situated on anionic surfaces, rendering the term APS somewhat misleading [4]. The principal targets of these antibodies encompass beta-2 glycoprotein-I and prothrombin [4]. For an APS diagnosis, patients must exhibit pregnancy morbidity, vascular thrombosis, or both, along with elevated titers of at least one of the following antibodies: lupus anticoagulant antibodies, anticardiolipin antibodies, or beta 2-glycoprotein-I antibodies [2]. The testing for these antibodies should occur on two separate occasions with a minimum interval of 12 weeks, and their levels should manifest as elevated in both assessments [2]. The diagnostic criteria for APS mandate the presence of at least one positive clinical manifestation coupled with at least one elevated laboratory titer [2]. Previously, APS treatment primarily relied on vitamin K antagonists, such as warfarin. Nonetheless, this approach was fraught with numerous complications and disease recurrence concerns [5]. Currently, the recommended therapeutic course entails the use of apixaban, a direct oral anticoagulant (DOAC) [5].

Patients may exhibit symptoms unrelated to thrombosis or pregnancy loss, presenting with psychiatric disorders, including psychosis, depression, mania, schizophrenia, or bipolar disorders [6]. Hallab et al. conducted a study that yielded the conclusion that hallucinations and delusions were the most frequently observed presenting symptoms among patients with APS-associated psychosis [7]. Given that psychosis can serve as the initial clinical manifestation of APS, it becomes imperative to incorporate APS into psychiatric education to enable early detection and mitigate potential comorbidities. In this context, a case report narrates the experience of a 31-year-old female who sought medical attention at the emergency department due to psychosis and subsequently suffered a thromboembolic stroke during her hospitalization.

## Case presentation

A 31-year-old Caucasian female presented to the hospital exhibiting erratic behavior in a public setting. Upon arrival in the emergency room, she demonstrated intense anger and agitation and even engaged in physical aggression toward nearby objects. Noteworthy physiological parameters at the time of assessment encompassed a blood pressure of 100/67 mmHg, a pulse rate of 99 beats/minute, a respiratory rate of 22 breaths/minute, and a temperature of 36.8°C. Following medical clearance, the patient was subsequently referred to the comprehensive psychiatric emergency program. During further inquiry, she attributed her behavior to the ingestion of K2 and an unspecified substance, manifesting uncooperativeness during the interview process. Of particular significance, the patient had a medical history marked by borderline personality disorder, post-traumatic stress disorder, and major depressive disorder. Notably, she was a daily tobacco cigarette user and consumed marijuana, cocaine, and K2. Furthermore, the patient exhibited an allergy to aripiprazole, trimethoprim-sulfamethoxazole, haloperidol, and penicillin (the latter aspect may be omitted as per preference). Initial laboratory results upon drawing revealed an elevated leukocyte count (10400/µL), a reduced hematocrit (35.9%), an increased prothrombin time (13.2 seconds), and increased partial thromboplastin time (48.0 seconds) (Table 1). For the management of psychosis, the patient was initiated on a regimen of risperidone 1 mg twice a day.

**Table 1:** A comparative assessment of the patient’s initial and most recent blood chemistry profile. ALT = alanine aminotransferase; AST = aspartate aminotransferase; WBC = white blood cell; NRBC = nucleated red blood cell; RBC = red blood cell; HBG = hemoglobin; HCT = hematocrit; MCV = mean corpuscular volume; PT = prothrombin time; INR = international normalized ratio; PTT = partial thromboplastin time

	Initial	Most recent	Latest reference range and units
Glucose	94	125 (H)	70–99 mg/dL
Blood urea nitrogen	14	10	7.0–17.0 mg/dL
Creatinine	0.62	0.66	0.52–1.04 mg/dL
Sodium	143	137	133–145 mmol/L
Potassium	4	3.6	3.5–5.1 mmol/L
Bilirubin total	0.6	0.6	0.2–1.3 mg/dL
ALT	22	18	<35 U/L
AST	20	20	14–36 U/L
Alkaline phosphatase	81	63	38.0–126.0 U/L
Albumin/Globulin ratio	1.9	1.5	-
WBC	10.4 (H)	11.1 (H)	4.5–10.2 × 10^3^/µL
WBC, corrected for NRBC	13.6	14.7	10^9^/L
RBC	4.2	3.11 (L)	3.95–4.83 × 10^6^/µL
HBG	12.4	8.9 (L)	11.4–15.5 g/dL
HCT	35.9 (L)	26.0 (L)	37–43.7%
MCV	85.7	83.6	82–94.5 fL
Platelets	227	236	180–401 × 10^3^/µL
PT	13.2 (H)	13.4 (H)	9.2–12.8 seconds
INR	1.18	1.19	0.70–1.20
PTT	48.0 (H)	107.6 (HH)	23.5–35.5 seconds

**Figure 1:**
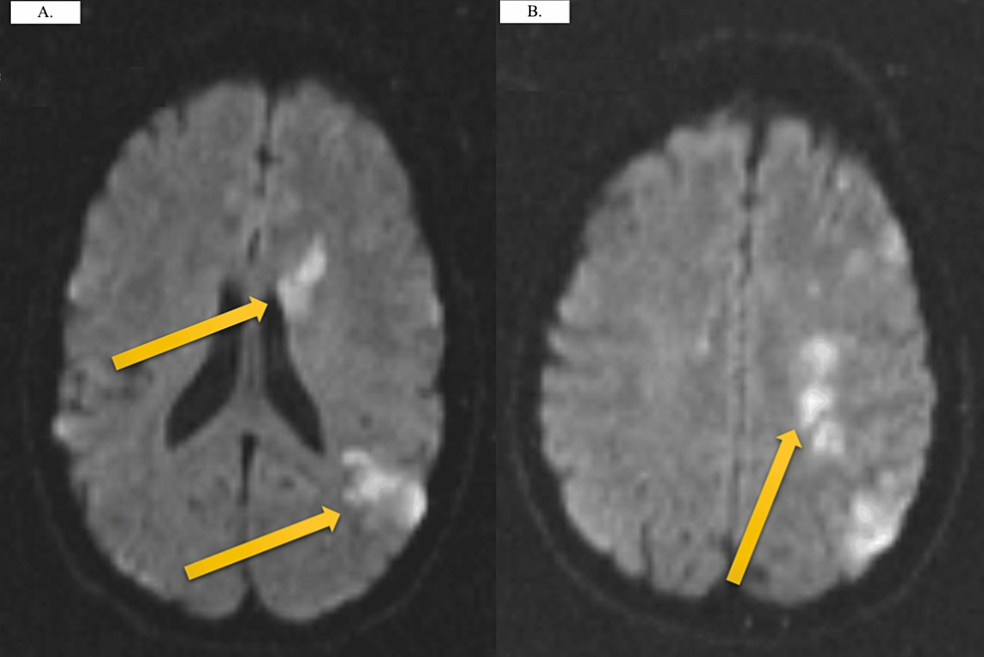
MRI demonstrating (A) acute left MCA, ACA, and watershed infarct (B) restricted diffusion within the distribution of the left MCA. Yellow arrows indicate regions of infarct. MCA = middle cerebral artery; ACA = anterior cerebral artery

On the second day of her admission, the patient was observed exhibiting restless movements and tossing in her bed, ultimately culminating in a fall to the floor. Interestingly, she did not report any pain or exhibit visible bruises following the incident. However, within a few hours, she required assistance to walk, displaying a leaning tendency toward the left side while standing. A comprehensive neurological examination further revealed right-sided facial drooping and weakness. As measured by the National Institutes of Health (NIH) stroke scale, her condition corresponded to a score of 5 (involving two points for level of consciousness questions concerning month and age, one point for minor facial palsy, one point for motor drift in the right arm, and one point for motor drift in the right leg). A CT scan of the head and neck without contrast did not indicate acute transcortical infarct, intracranial hemorrhage, or any mass effect (corresponding images acquired and interpreted on admission day two). Conversely, a CT angiogram of the head and neck with contrast highlighted the absence of stenosis or dissection in the neck and the brain but showed stenosis in the left middle cerebral artery (MCA) along with a sub-occlusive thrombus. To address her condition, the patient was initiated on a treatment regimen comprising aspirin 325 mg once, a maintenance dose of 81 mg twice daily through a nasogastric (NG) tube, supplemented by clopidogrel 300 mg once, and a maintenance dose of 75 mg four times a day through the NG tube. An EKG confirmed a normal sinus rhythm with a QTc of 366 ms (images available). Moreover, an MRI of the head without contrast, shown in Figure 1, demonstrated restricted diffusion within the distribution of the left MCA. These regions exhibited restricted diffusion on diffusion imaging and displayed low signal intensity on apparent diffusion coefficient imaging, consistent with acute ischemic changes (MRI imaging conducted on admission day two and interpreted on admission day three). However, imaging results were somewhat limited due to the patient’s movement during the examination. Subsequently, a Holter monitor assessment ruled out atrial fibrillation or atrial flutter as contributory factors. Following her brief stay in the psychiatric unit, the patient was transferred to the medical intensive care unit (MICU) to further manage her condition.

During the initial day in the MICU, the patient exhibited dilated reactive pupils and aphasia. The NIH stroke scale increased to 15, indicating a worsening of her neurological status. A CT scan of the head without contrast divulged infarction in the left frontal lobe, left parietal lobe, left caudate nucleus, and left basal ganglia, all classified as acute. Abnormal CT angiography results revealed decreased contrast opacification in the distal right M2 segments of the right MCA compared to the left. Concerning the left anterior cerebral artery (ACA), a short segment occlusion of the distal left A2 with minimal reconstitution of the distal segments was noted. Furthermore, patchy subcortical white matter hypodensities were evident in the bilateral frontal lobes and parasagittal frontal lobes. As such, the patient was referred to the interventional neurology unit for mechanical thrombectomy. Upon injection of the left common carotid artery, the presence of a thrombus at the origin of the left ACA and the left MCA was established. The MCA was found to be completely occluded, while the ACA was only partially occluded, allowing visualization of its branches with left leptomeningeal flow into the MCA territories. Although the left MCA thrombus removal was successful, attempts to extract the left ACA thrombus proved futile.

**Figure 2:**
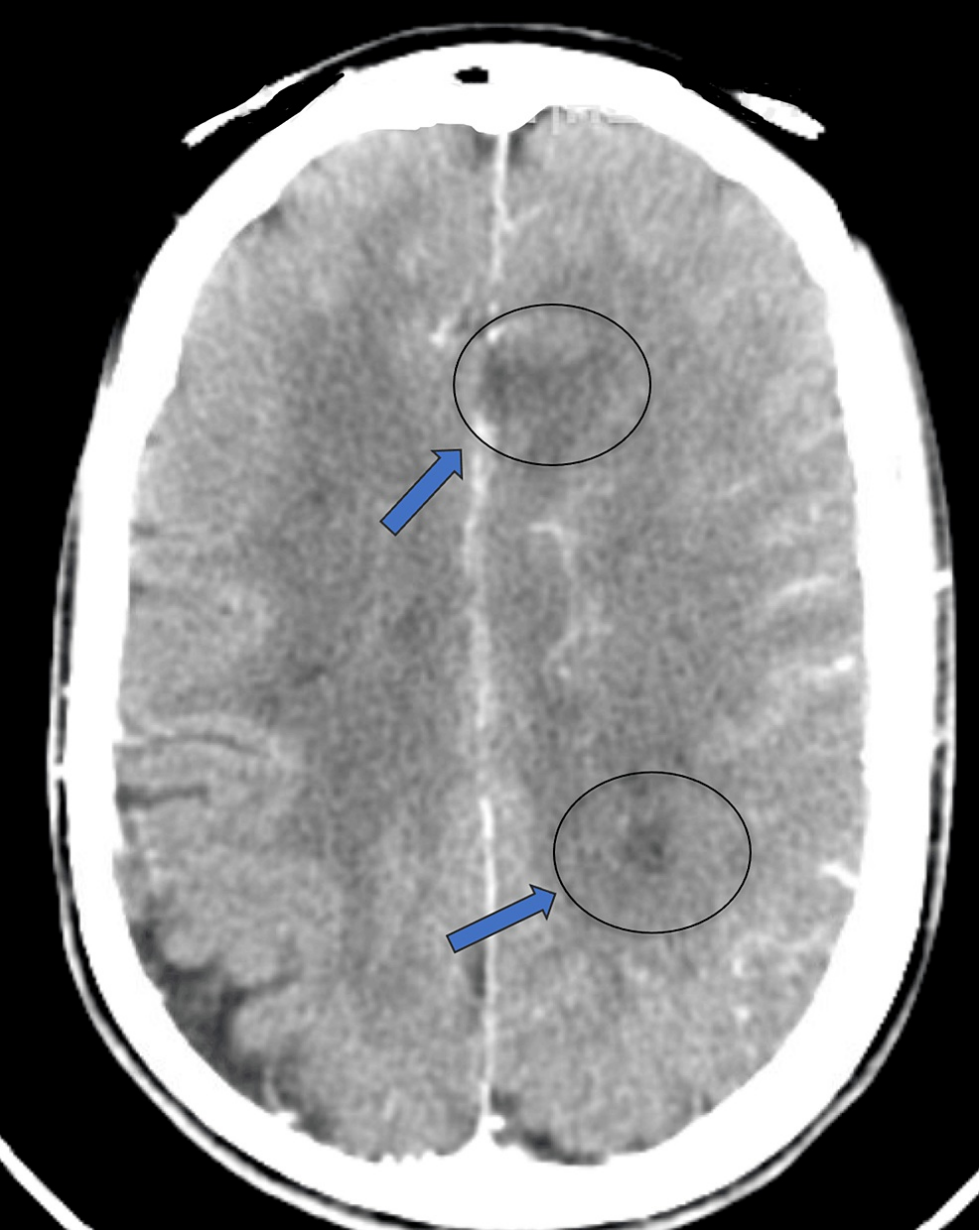
A follow-up CT scan conducted 48 hours post-mechanical thrombectomy indicating the presence of additional infarctions (blue arrows).

A post-procedure CT scan of the head without contrast exhibited a cytotoxic edema pattern in the left frontal lobe, left basal ganglia, and left posterior frontoparietal lobe regions, consistent with acute ischemic infarctions (Figure 2). A transthoracic echocardiogram indicated a left ventricular ejection fraction exceeding 70%, normal sizing in all four chambers, normal diastolic function, and patent foramen ovale. Considering a suspicion of hypercoagulable disorder, a panel of coagulation studies was initiated. The results of beta-2 glycoprotein I antibody IgG and anticardiolipin IgG yielded positive values (greater than 150 GPI IgG units and 66 U/mL, respectively) (Table 2). In light of these antibody findings, a diagnosis of antiphospholipid antibody syndrome was established. Four days following the procedure, a follow-up CT scan of the head without contrast demonstrated the expected evolution of the known left MCA territory infarct, with an augmented hypodensity at the left frontal, frontoparietal, basal ganglia, and posterior parietal regions. Lower bilateral Doppler ultrasound and upper right Doppler ultrasound did not reveal any clots, while upper left Doppler ultrasound identified clots in the proximal, mid, and distal forearm regions of the left basilic vein, alongside a clot at the elbow region in the cephalic vein. Multiple X-rays were performed during the patient’s hospital stay, although the results were considered insignificant.

**Table 2:** Patient’s lupus anticoagulation study profile. Ab = antibody; IgG = immunoglobulin G; IgM = immunoglobulin M; IgA = immunoglobulin A; INR = international normalized ratio, PTT = prothrombin time; dRVVT = dilute Russell venom viper time

	Most recent	Reference range
Anticardiolipin Ab, IgG	68	Negative: <15
		Indeterminate: 15–20
		Low to medium positive: >20–80
		High positive: >80
Anticardiolipin Ab, IgM	10	Negative: <13
		Indeterminate: 13–20
		Low to medium positive: >20–80
		High positive: >80
Beta-2 glycoprotein 1 IgG	>150	<20
Beta-2 glycoprotein 1 IgM	<10	<33
Beta-2 glycoprotein 1 IgA	11	<26
Prothrombin time	11.4	9.1–12
INR	1.1	0.9–1.2
PTT	33.4	22.9–30.2
dRVVT screen	63.8	≤47.0
dRVVT confirmation	37.2	-
DRVVT/Confirmation ratio	1.6	0.8–12
Hexagonal phospholipid neutralization test	20	0–11

Following a hospitalization period of 15 days, the patient was discharged with a prescription for apixaban 5 mg tablets to be taken orally every 12 hours for 30 days. Additionally, a single aspirin 81 mg tablet was recommended once daily, along with chlorpromazine 25 mg tablets to be taken orally three times a day, and quetiapine 25 mg tablets recommended for oral intake twice a day. Notably, the patient’s right-sided hemiplegia remained evident at the time of discharge. Ultimately, the patient was transferred to another hospital in a different state, as per the request of her family.

## Discussion

APS represents an autoimmune multifactorial disorder, characterized by the occurrence of arterial and venous thrombotic events, closely linked to the presence of at least one distinct autoimmune antibody [8]. The presence of specific antibodies such as anticardiolipin antibodies, beta-2-glycoprotein antibodies, or lupus anticoagulant antibodies is notably associated with APS [8]. Within APS patients, thrombosis emerges as the predominant contributing factor, leading to neurological manifestations encompassing cerebral venous thrombosis, cerebrovascular accidents, transient ischemic attacks, and strokes [8].

Aside from its well-documented thrombotic manifestations, APS exerts a broader impact on patients’ lives, affecting diverse organ systems and functions. Notably, valvulopathy, nephropathy, and neuropsychiatric symptoms represent additional facets of APS pathology, contributing to the overall disease burden [9]. Of particular concern is the cognitive domain, as APS has been associated with cognitive impairment, particularly affecting verbal fluency and complex attention, as elucidated in a review conducted by Asif et al. [8]. Within the population of primary APS patients, cognitive impairment is prevalent in 42%-80% of cases [8]. Moreover, Erkan et al. revealed a noteworthy linear correlation between the levels of antiphospholipid antibodies and the severity of cognitive impairment, underscoring the potential role of antiphospholipid in the cognitive pathophysiology of APS [10]. Such findings further emphasize the significance of addressing cognitive issues in the management and care of APS patients.

In the realm of primary APS, a notable clinical feature may involve the occurrence of psychosis, a phenomenon well-documented in the medical literature [8]. Despite extensive research, a precise etiology linking APS to psychosis remains elusive [8]. The first case report documenting an association between antiphospholipid and psychosis emerged in 1994, wherein a 50-year-old woman initially presented with a schizophrenia-like syndrome, subsequently attributed to APS [11]. This compelling case exemplifies the intriguing potential for psychosis to precede the discernible physical manifestations commonly observed in APS patients [11].

Various risk factors have been identified in APS patients presenting with psychosis, encompassing female sex, a later onset of symptoms (with a mean age of 35 years), abrupt onset of the psychiatric condition, and the presence of hallucinations and delusions [7]. Notably, Asif et al. assert that APS-related delusional presentations are more likely to manifest with psychosis, with hallucinations being the most frequently encountered psychotic symptom [8]. Hallab et al. also elaborate on the spectrum of psychotic symptoms observed in APS patients, which may include auditory and visual hallucinations, paranoid ideation, and, on occasion, catatonia [7]. The pathophysiological mechanisms underlying APS-associated neuropsychiatric manifestations involve antiphospholipid-mediated disruptions at three distinct stages, namely, disturbance of cell membrane hemostatic reactions, heightened procoagulant activity due to cellular stimulation, and direct damage to neuronal pathways. These intricate processes contribute to the development of neuropsychiatric complications in APS patients [8]. Regrettably, the lack of specific diagnostic tests poses challenges in distinguishing primary from secondary psychosis in the context of APS, rendering the differentiation an area of clinical complexity [12]. As research advances, a deeper understanding of the underlying mechanisms will be vital for improved diagnosis and targeted interventions for patients grappling with APS-associated psychosis.

In a significant study conducted by Kao et al., encompassing a cohort of 22 female APS patients, it was observed that these individuals experienced neuropsychiatric manifestations despite presenting with normal MRI results [13]. As delineated by Asif et al., depression emerges as the most prevalent neuropsychiatric complication associated with APS, exhibiting the potential to manifest weeks to months before the onset of the initial thrombotic event [8]. The pathogenesis of such mood changes is likely attributed to the impact of anticardiolipin antibodies on serotonergic and dopaminergic neurons [8]. In the context of APS, depression can either manifest concomitantly with psychosis or may present as an independent entity [8]. Furthermore, an investigation conducted by Maes et al. demonstrated that a considerable proportion of patients diagnosed with depression also tested positive for antiphospholipid antibodies, underscoring the potential link between depression and the autoimmune milieu characteristic of APS [14]. These findings add to the growing body of evidence supporting the intricate relationship between APS and neuropsychiatric complications, necessitating careful attention to mental health concerns in the management of APS patients.

APS exhibits the potential to give rise to dementia, which may even serve as an initial presenting symptom in affected individuals [8]. Given this consideration, patients presenting with acute and severe dementia should undergo evaluation for possible underlying autoimmune diseases such as APS [8]. Remarkably, a study conducted by Sorice et al. demonstrated the beneficial impact of APS treatment on dementia, hinting at the reversible nature of cognitive impairments in the context of APS [15]. Moreover, the same investigation revealed a noteworthy association between positive anticardiolipin antibodies and diminished cognitive function, as opposed to patients exhibiting negative anticardiolipin antibodies [15]. This intriguing correlation sheds light on the role of specific autoantibodies in the development and progression of cognitive decline within APS patients, offering valuable insights into the management of dementia in this clinical context.

Due to the scarcity of comprehensive data on APS patients deviating from standardized diagnostic criteria, a consensus on optimal treatment strategies remains elusive [9]. Treatment options include using DOACs, vitamin K inhibitors, heparin, or antiplatelet agents [16]. This case report illustrates an instance wherein a patient initially presented with psychotic symptoms, eventually linked to APS. Given the unique clinical context, further investigation is imperative to elucidate the impact of APS on the psychiatric well-being and stability of affected individuals. A deeper understanding of these interactions holds the potential to inform more targeted and effective interventions for APS patients with psychiatric manifestations.

## Conclusions

Neuropsychiatric symptoms in patients with APS encompass a diverse range of manifestations, varying from focal deficits to global dysfunction. While strokes and transient ischemic attacks are common neurological presentations in APS, a broad spectrum of other neurological features, including non-thrombotic syndromes, have been documented in association with the presence of antiphospholipid antibodies. The recognition of APS has significantly impacted the understanding and management of central nervous system manifestations, warranting tailored approaches to treatment based on the specific neurological presentation. As APS remains a complex and multifaceted condition, further research is imperative to unravel the underlying mechanisms linking APS to neuropsychiatric symptoms, enabling improved patient care and outcomes in this intricate domain.
